# A pilot study into reaching performance after severe to moderate stroke using upper arm support

**DOI:** 10.1371/journal.pone.0200787

**Published:** 2018-07-17

**Authors:** Matthew R. Williams

**Affiliations:** 1 Louis Stokes Cleveland VA Medical Center, Cleveland, OH, United States of America; 2 Cleveland FES Center, Cleveland, OH, United States of America; 3 Department of Biomedical Engineering, Case Western Reserve University, Cleveland, OH, United States of America; University of Illinois at Urbana-Champaign, UNITED STATES

## Abstract

Stroke effects millions of people each year and can have a significant impact on the ability to use the impaired arm and hand. One of the results of stroke is the development of an abnormal shoulder-elbow flexion synergy, where lifting the arm can cause the elbow, wrist, and finger flexors to involuntarily contract, reducing the ability to reach with the arm and hand opening. This study explored the effect of using support at the upper arm to improve hand and arm reaching performance. Nine participants were studied while performing a virtual reaching task under three conditions: while the weight of their impaired arm was supported by a robot arm, while unsupported, and while using their non-impaired arm. Most subjects exhibited faster and more accurate reaching while supported compared to unsupported. For the subjects who could voluntarily open their hand, most were able to more swiftly open their hand when using upper arm support. In many cases, performance with support was not statistically different than the unaffected arm and hand. Muscle activity of the impaired limb with upper arm support showed decreased effort to lift the arm and reduced biceps activity in most subjects, pointing to a reduction in the abnormal flexion synergy while using upper arm support. While arm support can help to reduce the activation of abnormal synergies, weakness resulting from hemiparesis remains an issue impacting performance. Future systems will need to address both of these causes of disability to more fully restore function after stroke.

## Introduction

Stroke is a common occurrence in the U.S.; Approximately 795,000 Americans suffer a stroke every year [1]. It is the third leading cause of death and one of the main causes of disability. There are currently 7,000,000 chronic stroke survivors over 20 years old in the U.S., representing about 3% of the general population [1]. In the veteran community, over 5,000 veterans are hospitalized each year due to ischemic stroke, with those patients accounting for over 10% of the case load and costing more than three times the overall average [2]. Recent studies have shown that there is a significantly increased risk of stroke in people who have suffered traumatic brain injuries (TBIs) [3,4] and in patients with Post Traumatic Stress Disorder (PTSD) who are often on potent antipsychotic medications [5]. With these conditions being seen in remarkably greater numbers in the current military engagements compared to previous combat actions, there exists the likelihood of the VA seeing progressively more stroke survivors [6,7].

There are many potential effects of a stroke, depending on where in the brain the event occurred. Approximately 50% of stroke survivors over age 64 have some hemiparesis affecting control of the arm and hand [1] with the vast majority (88.4%) not regaining complete function [8]. Moderate to severe hemiparesis can have a significant impact upon many common activities of daily living (ADLs), resulting in significant dependence on caregivers. In particular, upper limb hemiparesis, which occurs in approximately 26% of stroke survivors [1], negatively impacts bimanual tasks, such as opening containers, cutting food, and holding open a bag such as a wallet or grocery bag. In addition to hemiparesis, stroke survivors can also develop abnormal muscle synergies where voluntary effort to contract the muscle or group of muscles needed to execute a task causes other muscles not normally involved in the task to involuntarily contract, resulting in loss of control or coordination during certain movements [9–14].

One common abnormal synergy is the shoulder-elbow flexion synergy where the elbow, wrist, and fingers flex involuntarily when the patient abducts or raises the shoulder–resulting in a loss of reach area and difficulty performing ADLs. The magnitude of this effect is related to the amount of effort the individual exerts. Abnormal synergy does not occur if the arm is manually lifted by an outside force (e.g. by a therapist or assistive device). However, when the individual lifts their arm voluntarily, the synergy is activated, with increasing amounts of shoulder abduction torque resulting in greater elbow, wrist, and finger flexion torque, which reduces the overall reach volume of the arm [9,12].

In studies of individuals with stroke, providing gravity compensation at the forearm has allowed participants to access a greater range of motion. In the case of stroke this is the result of reducing abnormal muscle synergies [9,15], and allowing for retained voluntary control to be more effective.

One way to provide this type of support is to use a mechanical assistance device. Over the years, a number of robots intended for post-stroke rehabilitation of the upper extremity have been developed for both therapy and to assist daily function. Notable examples of therapy devices include the ARMin [16–18] and RUPERT [19,20]. The MIT MANUS robot has been clinically evaluated and has shown statistically significant although functionally modest results in rehabilitation and motor re-learning [21]. While these previous devices were lab-based robots intended for therapy, a number of robotic devices for assisting arm function as a form of “force prosthesis” have been developed that range from devices to support the arm against gravity [22–26] to full arm, powered exoskeletons [27,28]. The primary drawback to these machines is that due to the force requirements and the kinematics of applying force assistance at the end of the forearm, they tend to be large and are required to be mounted to a user’s wheelchair or other rigid structure [29]. While some stroke patients are limited in terms of walking mobility, a large number are ambulatory and do not require a wheelchair, and hence are not interested in using these large, primarily lab-based mechanical devices to assist in their daily activities. For these users, a different solution is needed.

Thus far, all of the devices in clinical use attempt to reduce abnormal muscle synergy by providing gravity assistance at the forearm, but support at the upper arm, between the shoulder and elbow, has not been explored. This work investigates the hypothesis that upper arm support can assist shoulder abduction by producing gravity compensation for the affected limb and improve reaching capacity.

## Methods

To explore the impact of upper arm support on improving reaching and hand opening, research participants were asked to perform directed reaching tasks in a virtual reality environment. This was done over three conditions, 1) with their impaired arm while supported, 2) with the impaired arm without support, and 3) with their unaffected arm as a “gold standard” for comparison.

### Participants

For this work, nine people who have suffered a severe to moderate stroke (Table 1) were recruited through the stroke research programs in cooperation with clinicians at the Louis Stokes Cleveland DVA Medical Center (LSCDVAMC). Inclusion criteria consisted of: 1) being greater than 6 months post-stroke, 2) age between 20 and 80 years old, 3) having paresis confined to one side of the body with upper limb motor impairment, and 4) presenting moderate to severe impairment (Fugl-Meyer upper limb assessment between 15 and 47) including a reduced reach volume and reduced voluntary extension of the joints of the affected arm. Criteria for excluding participants was: 1) having substantial pain in the impaired limb, 2) having sensory impairment of the affected limb, 3) having visual deficits beyond those that can be corrected with corrective lenses, 4) being unable to perceive or visually track objects shown on a computer screen, 5) exhibiting cognitive impairment that would preclude the individual from following simple instructions similar to those common to standard of care therapy practices, and 6) having apraxia or significant neglect of the impaired limb. All subjects were able to give written Informed Consent and all research protocols were approved by the Louis Stokes Cleveland Department of Veterans Affairs Medical Center Institutional Review Board, IRB #16050-H37. Prior to any reaching experiments, participants were screened using the Mini Mental State Exam to verify that they were cognitively capable of both providing Informed Consent as well as able to follow basic instructions.

**Table 1:** Participant demographics.

			Time Post		Upper Ext.
	Gender	Age	Stroke	Aff.	Fugl-
Participant	(M/F)	(yr)	(yr)	Limb	Meyer
1	M	56	12	R	23
2	M	56	3	R	36
3	M	63	11	R	34
4	M	58	5	R	37
5	F	31	5	R	20
6	F	60	2	L	18
7	M	73	5	R	31
8	M	62	12	R	27
9	M	77	9	L	46

### Upper arm support

**Fig 1:**
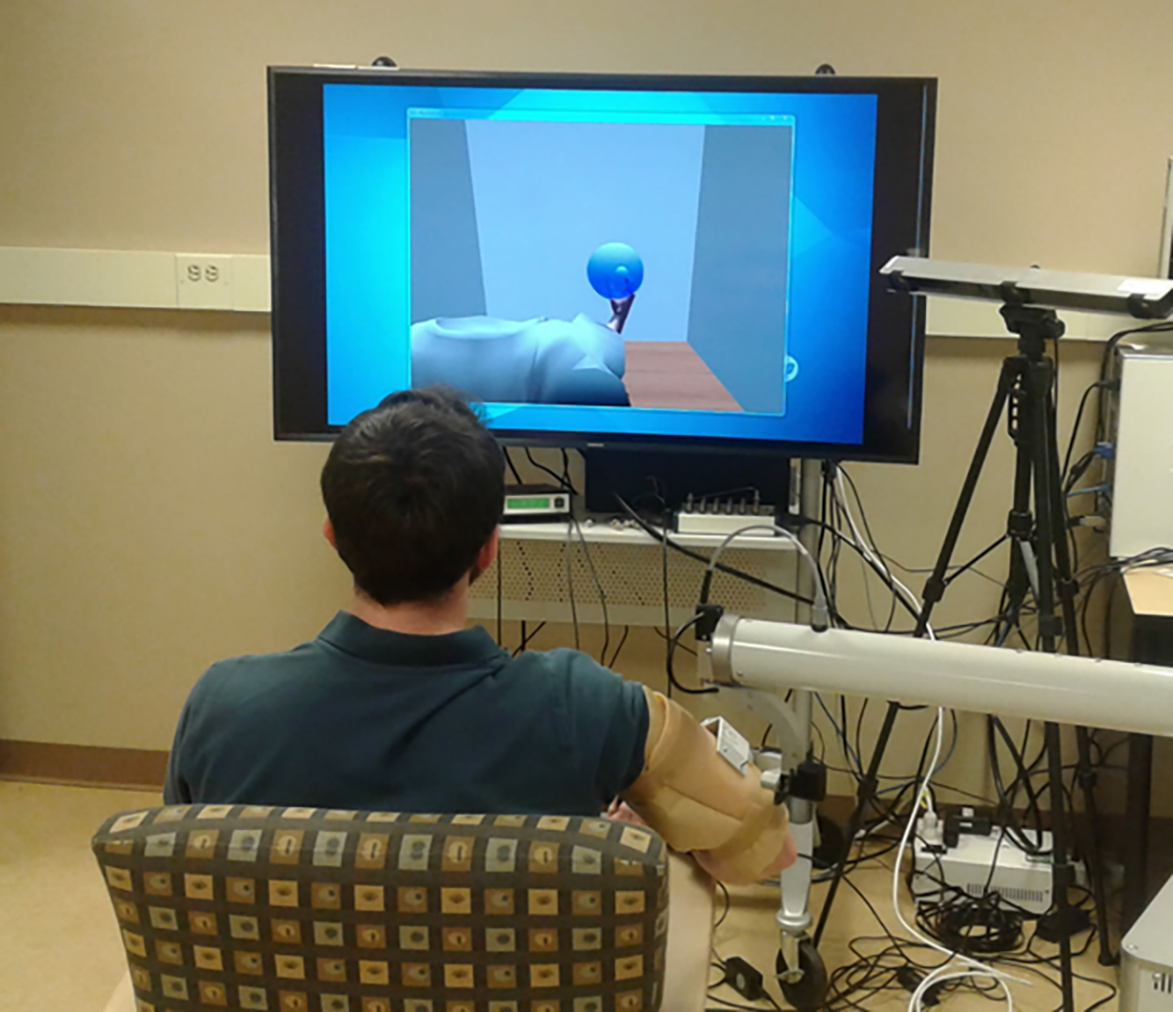
Photograph of experimental set-up showing VR display, motion capture camera, arm support, and subject. The individual in this manuscript has given written informed consent (as outlined in PLOS consent form) to publish these case details.

For this work, some reaching tasks were conducted while the impaired arm was supported between the shoulder and elbow by a HapticMaster (Moog, FCS, Netherlands) robot arm via a molded arm orthosis and a 3D gimbal (Fig 1). The robot was programed to provide gravity compensation of the arm such that the weight of the limb (as perceived by participants) was effectively zero. Each participant’s limb was secured to the robot and weighed using the onboard sensors. This weight was then scaled up by 50% (determined in pilot experiments) to achieve a “neutral” gravity assistance for the arm.

### Reaching task

Participants performed a “center-out” target matching task where they moved their hand from rest to a target locations within a virtual reality (VR) environment. Participants were seated in front of a large computer monitor (Fig 1) displaying a 3D animation of a hand and arm in a VR workspace from a first person view (GameStudio Conitec DataSystems, Inc. La Mesa, CA). Participants wore a cluster of IR reflective markers on a wrist brace and the position of the end of their arm was recorded using an Optitrak V120: Trio motion capture system (Natural Point, Inc., Corvallis, OR). This endpoint position was passed to a Simulink model (Mathworks, Natick, MA) that controlled the input to the VR visualization such that the VR arm mimicked the position and motion of the recorded physical limb [30]. Participants were given 5–10 minutes of “free run” practice to become accustomed to the system by performing non-guided, self-selected reaching actions.

**Fig 2:**
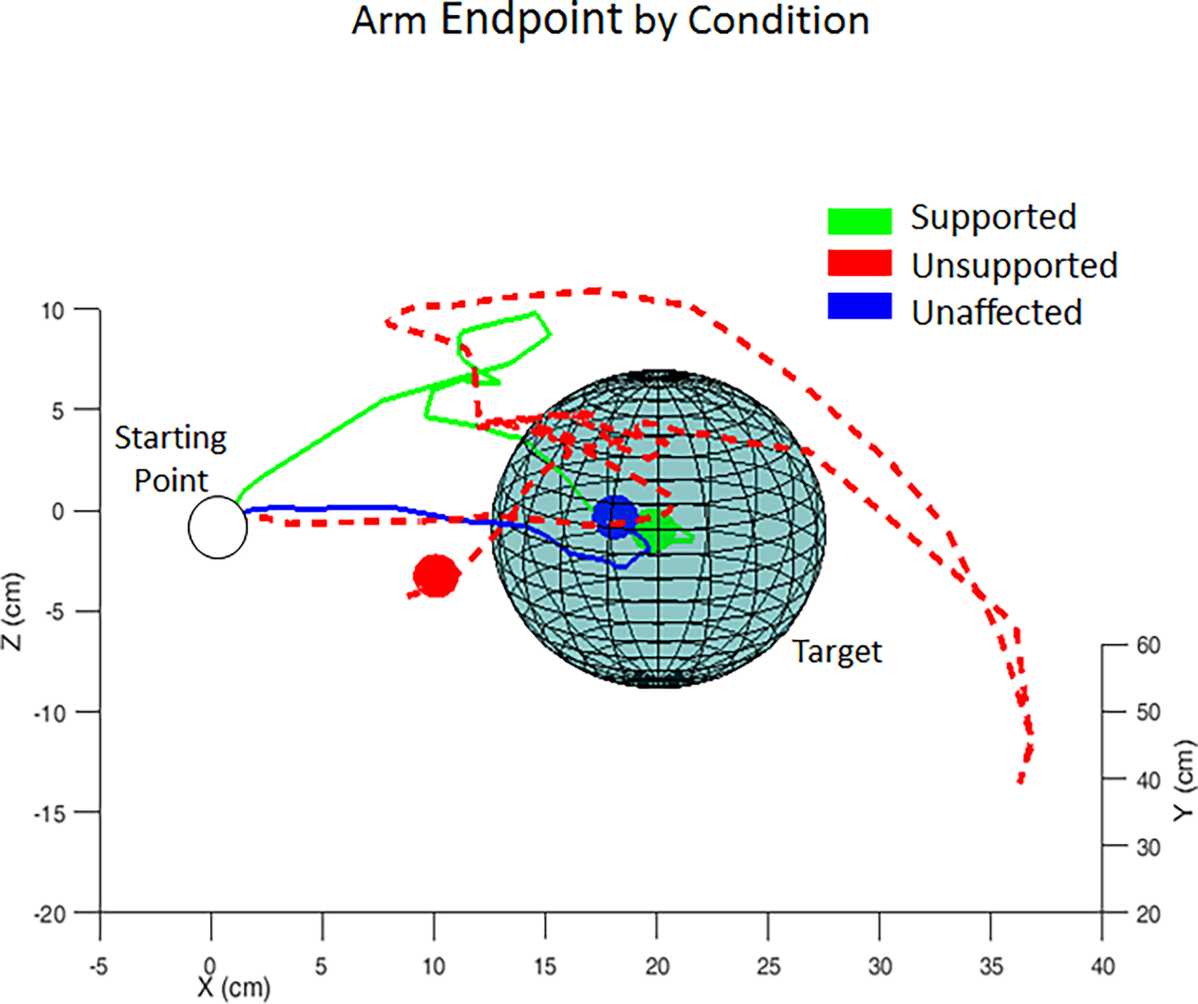
Reach target locations centered around hand starting position at rest. Numbers to the right of each marker are the percentage of subjects who were able to reach that target with Support that could not while Unsupported.

For the experiment, spherical targets were presented at specific fixed distances and directions from the starting point (Fig 2). Participants were instructed to begin each trial with their hand resting on the armrest which set the starting point for the trial. This dynamic calibration of the targets relative to the starting point of the arm allowed for better comparison across reaches by eliminating the variability of having to begin at an exact starting location. Targets were located 10 and 20 cm from the starting point and were arranged to require varying degrees of arm extension to be reached. Most targets (5 of 7 at each distance) were in the horizontal plane of the starting point, however one was directly upward and one was backward and upward toward the chest. These 14 total target locations cover reaches that would be needed to perform most activities of daily living (ADLs) while seated. Targets were shown to participants for 5 seconds to allow for reach planning and were then cued to move. Reaches were cued to begin by using colored targets–white to indicate target location prior to moving, green to move to the target, red when in the target zone and they should select the target, and blue when the task was complete. The target space was a sphere of 15 cm diameter.

Targets were selected in one of two ways. For those participants who could voluntarily open their hand (at least 25% of full finger extension, four of nine participants), an electrogoniometer (F35, Biometrics, Inc., UK) was taped over the first joint of the finger showing the most extension (index or middle). Prior to starting the experiment, the participant’s maximum and minimum voluntary hand opening was measured. To select targets, these participants had to exhibit at least 50% hand opening (maximum–minimum) while in the target zone. Because the inclusion criteria included individuals with severe to moderate stroke, some participants (five of nine) were unable to voluntarily open their hand due to their level of impairment. For those who could not open their hand, participants had to maintain the VR hand within the target zone for 1 s to select the target. While this method is different from the less impaired participants, the difference in performance is noted in the results. As the overall goal of the work is to show any differences in reaching performance, the difference in target selection is not seen as an overly disruptive confound and differences between participant groups are discussed.

Differential surface EMG recordings were recorded from five arm and shoulder muscles using standard self-adhesive quad-polar Trigno electrodes (Delsys, Inc., Natick, MA) placed over the muscle belly of each muscle and transmitted to a PC running xPC Target (Mathworks, Natick, MA) and equipped with a data acquisition card (PCI-6259 M, National Instruments, Austin, TX). The analog signals were sampled at 2.5 kHz and band-pass filtered between 20 and 500 Hz for analysis. EMGs were be recorded from the anterior and middle deltoid, trapezius, biceps, and triceps. The electrodes were not disturbed and were kept in place for both the supported and unsupported conditions.

Participants performed each set of 14 reach trials repeated in two blocks per condition. Condition order was randomly determined for each participant. Participants were permitted to rest as they felt comfortable between each reaching task and were required to rest for 10 minutes between each condition.

### Performance measures

A series of previously developed performance measures were used to assess each participant’s reaching performance across conditions [31]. These included: 1) *Throughput*, a summary of overall target reaching performance; 2) *Path Efficiency*, a measure of path straightness to the target; 3) *Overshoot*, the number of times participants entered and exited the target zone without selecting it; and 4) *Movement Time*, the time to reach the target and select it (not including the required dwell time). For those subjects who could open their hand, the performance of that action was evaluated by computing their *Time On Target* which was determined as the proportion of the trial time they spent in the target zone such that faster hand opening would result in a shorter *Time On Target* in relation to the total reach time.

The mean of the EMG amplitude for each muscle was computed and averaged across both trials for the supported and unsupported conditions. Changes in muscle activity were assessed based on the number of participants who showed reduced mean EMG amplitude while using arm support compared to without support for each reach target. To explore the impact of arm extension on EMG, the reduction in average EMG amplitude between the supported and unsupported conditions was ordered for each horizontal reach direction for those requiring the least to the most extension. The Pearson correlation was then found between these ranked values and the amount of extension required (1 to 5, with 1 requiring the least and 5 the most extension)

To capture participants perception of how arm support affected their reaching, after the experiment they were asked to rate their performance both while using arm support and without using the Usability Rating Scale (URS), [32,33] an instrument ranging from “very difficult” (-2.5) to “very easy” (+2.5). Subjects rated their ability to reach the targets, control the position of the hand, open the hand, and the overall ease or difficulty of the task with and without support.

### Statistical analysis

Performance measures by condition for each subject were averaged over both reaching trials for all targets they could reach in both trial blocks. Normality of the data was determined using graphical techniques. Assessment of performance across conditions both within and across subjects was evaluated using one way ANOVA with secondary analysis using Tukey’s Honestly Significant Difference test to compare between conditions. Statistical differences in perception of difficulty were determined by application of the Wilcoxon Signed Rank Sum test to the URS data. Significance of EMG mean amplitude reduction correlation to reach direction was determined as per [34]. In all cases, p≤0.05 was considered significant.

## Results

**Fig 3:**
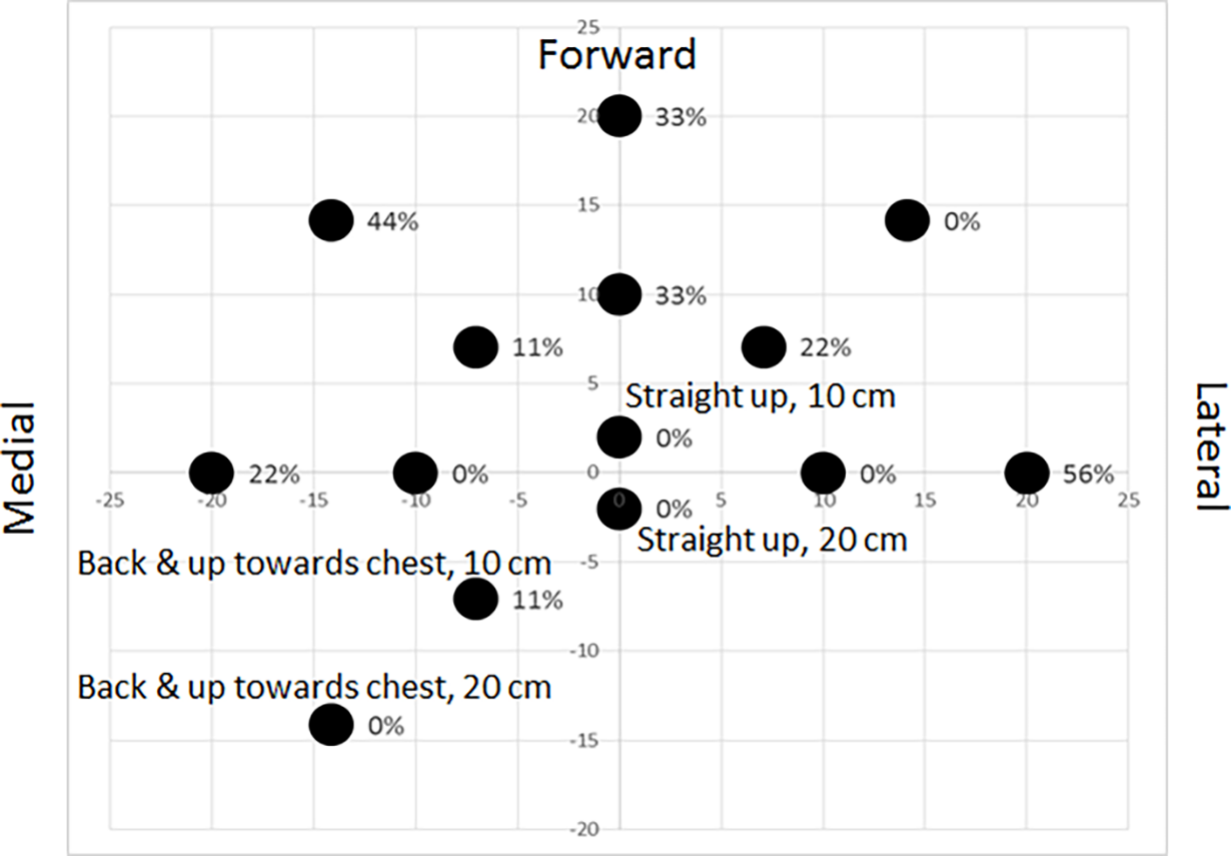
Example of typical reach. The reach shown is to a target lateral to the arm. The dashed trace of the unsupported limb indicates that the subject was unable to successfully acquire the target. The blue Unaffected arm trace has been mirrored over to the impaired side for illustrative purposes. The starting point is indicated with a white ball and the end points of each reach are shown by a ball matching the color shown in the legend for each condition.

The arm endpoint path for a typical reach of the impaired arm while supported, unsupported, and the unaffected arm can be seen in Fig 3. This figure shows an example of the change in end point control of the impaired limb compared to the unaffected arm and illustrates the difference upper arm support can have in the reaching task.

Across all participants, support impacted the ability to successfully reach and select targets, particularly those father from the central starting point with reduced improvement to the closer targets (Fig 2). Lateral reaches were most improved with support with the other targets requiring greater amounts of extension also benefitting from arm support. The target requiring the greatest amount of extension (forward and lateral) did not any improvement with support.

**Fig 4:**
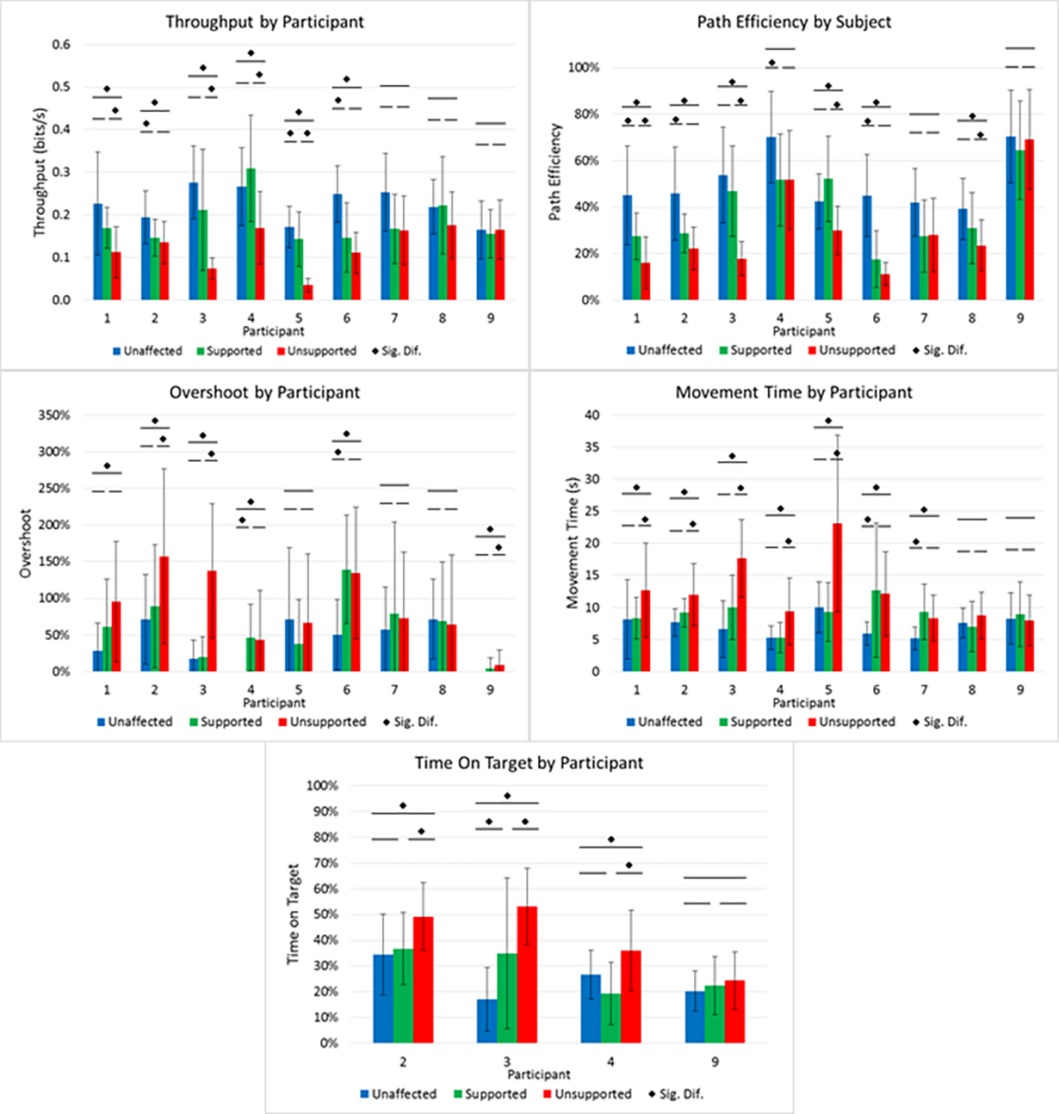
Reaching performance by participant across all measures.

**Fig 5:**
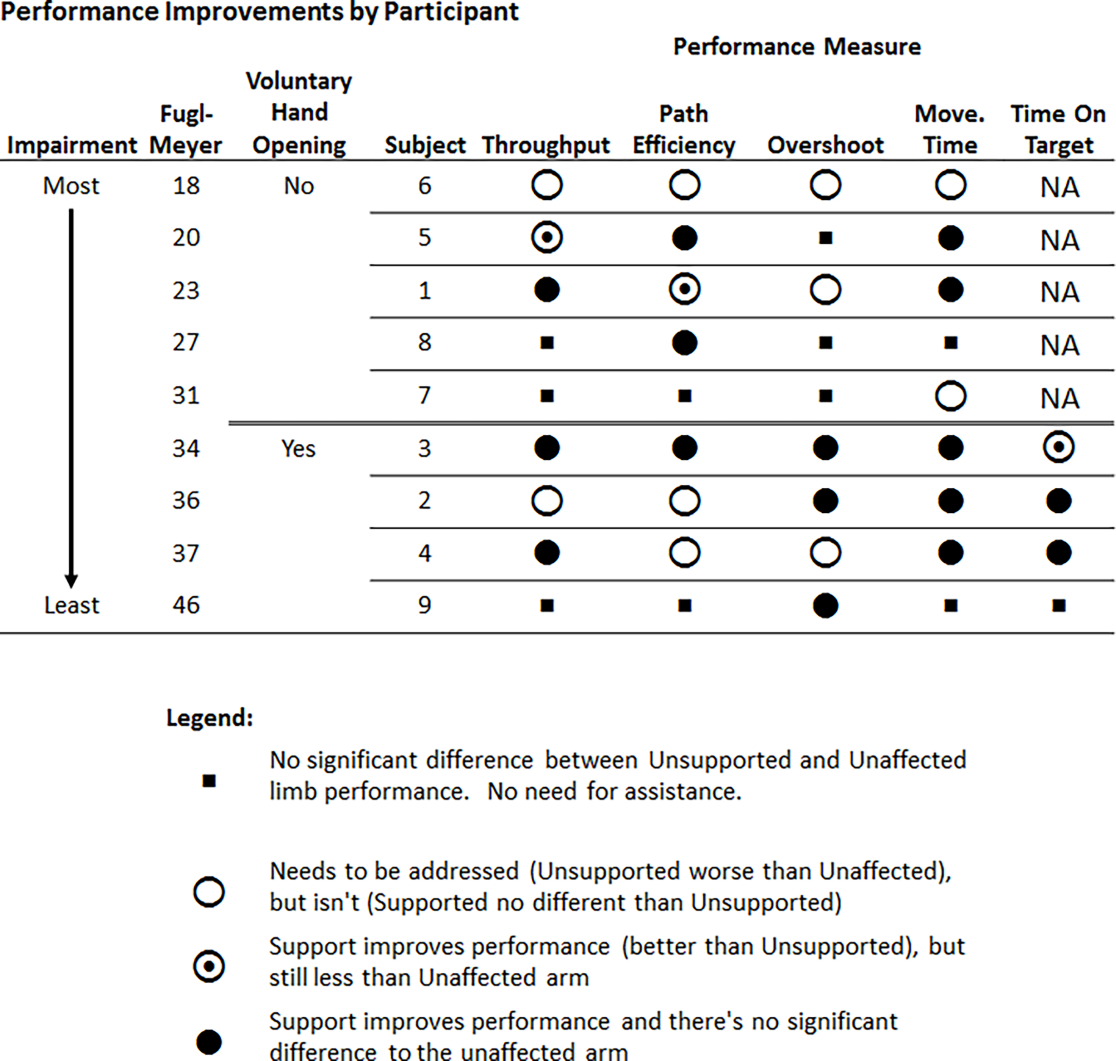
Summary of performance improvements by participant, ranked by level of impairment. Most participants showed significant improvements in Movement Time with some also improving their Throughput and Path Efficiency while using upper arm support compared to without support.

Individual subject performance across all metrics is shown in Fig 4 with a summary of improvements due to upper arm support in Fig 5. Improvement was assessed on the basis of 1) the number of subjects who showed significantly worse performance in the impaired arm compared to the unaffected limb, 2) the number of subjects who showed significant improvement between the supported and unsupported conditions and 3) the number of subjects for whom there was no statistical difference between the impaired arm while supported and the unaffected arm. For Throughput, of the six participants who exhibited reduced impaired arm performance compared to unaffected, four demonstrated significantly higher Throughput while supported and three of those had no significant difference between supported and unaffected (Figs 4 and 5). Of the seven who demonstrated less Path Efficiency with the impaired arm compared to unaffected, four displayed significant higher Path Efficiency while supported and three of them had no significant difference to unaffected Path Efficiency (Figs 4 and 5). Overshoot was less affected by arm support with three participants exhibiting a significantly reduced number of Overshoots while using support over unsupported out of a total of the six participants who expressed statistically higher Overshoot in the impaired arm compared to unaffected (Figs 4 and 5). For the seven participants who had a significantly higher Movement Time using the impaired arm compared to the unaffected limb, five had significantly lower Movement Time while supported and all five showed no significant difference in Movement Time between the supported and unaffected conditions (Figs 4 and 5). Of the four participants who had voluntary hand opening, three had a statistically higher Time On Target with the impaired arm compared to the unaffected limb of which all three had a significantly lower Time On Target while supported and two of those expressed no significant difference in Time Of Target between supported and unaffected (Figs 4 and 5).

**Fig 6:**
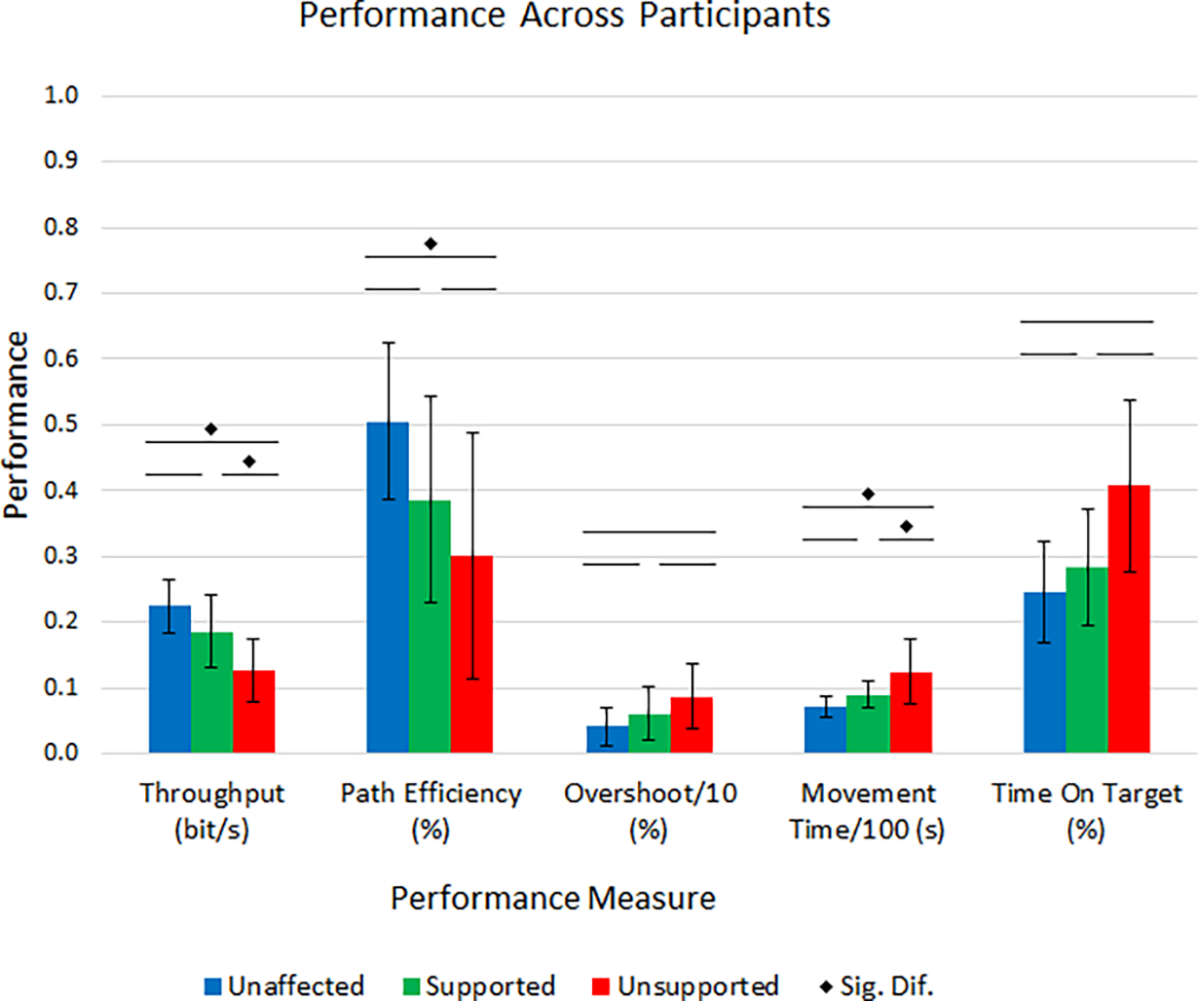
Average performance across all participants. Significant population improvements were observed in both Throughput and Movement Time due to using upper arm support over unsupported. In all measures, there was no significant difference between the performance of the supported impaired arm and the unaffected limb. This is contrasted by the significantly reduced performance of the unsupported impaired arm compared to the other limb.

Across all subjects, the average Throughput was significantly higher and Movement Time significantly lower for reaches with the impaired limb with support than without (Fig 6). For all performance measures except Overshoot and Time On Target the unaffected arm had statistically superior performance compared to the unsupported impaired limb and no significant difference in performance compared to the supported impaired limb. Average impaired limb Path Efficiency across subjects while supported was not statistically different than without. No statistical differences were observed in the average Overshoot or Time On Target across conditions.

**Fig 7:**
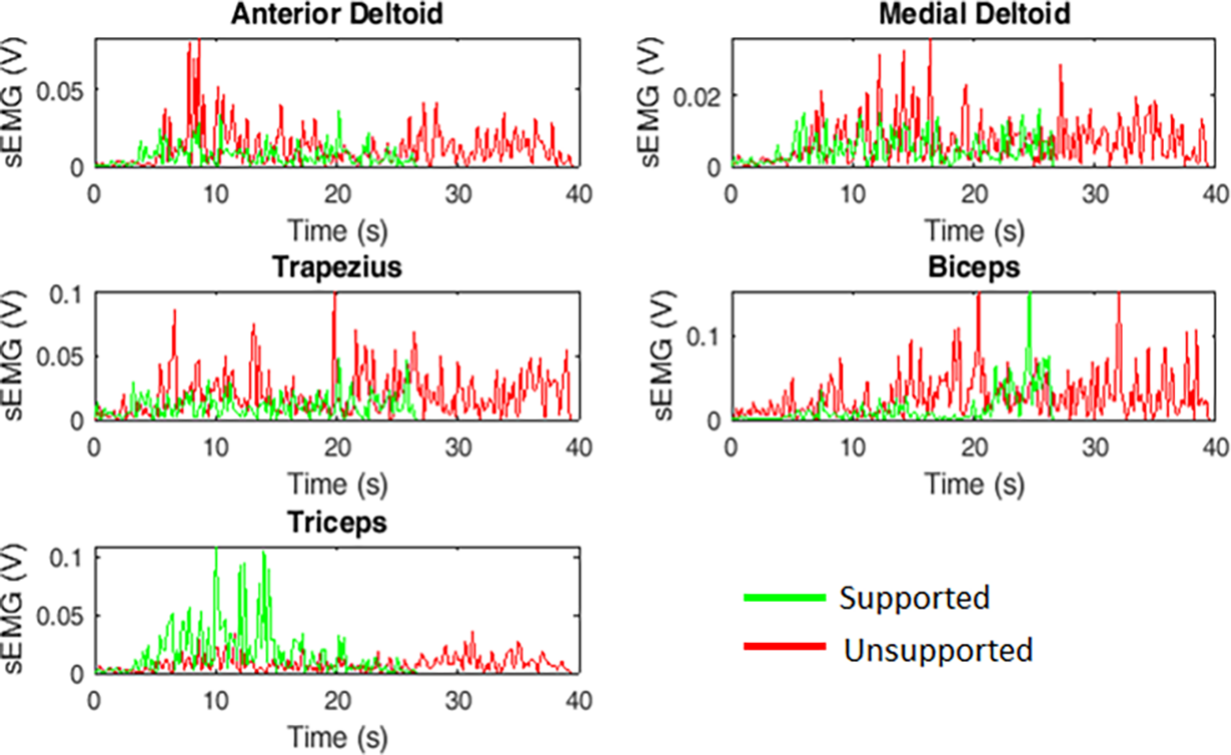
Example of EMG activity during reaching task. In this figure, the participant is reaching laterally to the far target.

Most participants exhibited reduced muscle activity when using arm support as shown in the representative EMG plots in Fig 7. The proportion of participants who had lower mean EMG activity while using upper arm support for each type of reach is shown in Table 2. Most subjects exhibited reduced activity in the anterior deltoid and trapezius with support, with fewer subjects showing reduced mean activity in the other muscles across all reaches. With respect to the type of reach, the number of subjects who reduced their mean medial deltoid activity as a function of extension was significantly negatively correlated. For the impaired biceps, the number of subjects who exhibited reduced muscle activity with increased extension was significantly positively correlated such that the more extension that was required to reach a target, the more subjects had reduced average biceps activity while using support than without. No other similar trends were seen in the other muscles.

**Table 2:** Proportion of participants exhibiting reduced mean muscle activity while supported by reach target direction.

		Target Direction					
	Target		Medial-			Lateral-	
Muscle	Distance	Medial	Forward	Forward	Lateral	Forward	r
Ant. Deltoid	10 cm	100%	89%	78%	89%	100%	0.00
	20 cm	78%	100%	89%	89%	100%	0.57
Med. Deltoid	10 cm	89%	67%	67%	67%	56%	-0.87
	20 cm	78%	67%	56%	56%	56%	-0.88
Trapezius	10 cm	100%	100%	89%	100%	100%	0.00
	20 cm	100%	100%	100%	100%	100%	0.00
Biceps	10 cm	44%	67%	78%	89%	100%	0.99
	20 cm	44%	78%	67%	89%	100%	0.90
Triceps	10 cm	67%	56%	67%	67%	78%	0.67
	20 cm	67%	56%	67%	56%	67%	0.00
		**Least**	**←Amount of Extension Required→**			**Most**	

The average difference in user perception in the ease of reaching targets with the impaired arm between the supported and unsupported cases is shown in Table 3. Significant positive differences were expressed in the ease of reaching to targets, controlling hand position and performing actions while supported over unsupported. No statistical difference in ease of hand opening was observed between supported and unsupported.

**Table 3:** Differences in participant reported URS scores for four general actions.

Difference between conditions	Participant											
(Supported—Unsupported)	1	2	3	4	5	6	7	8	9	Mean	Std Dev.	p
Reaching to targets	0.5	3.5	0	2.5	4.5	0.5	4	1	0.5	1.89	1.75	0.007*
Controlling hand position	1	2	0.5	2.5	4	0.5	2	1	3.5	1.89	1.27	0.006*
Opening hand	0	0	0.5	0.5	0	0	1	0	3.5	0.61	0.38	0.437
Performing actions	1.5	0.5	0	1.5	3.5	2.5	1	2	3.5	1.78	1.12	0.009*
Reduced Rating		-5	-2.5	0	2.5	5	Improved Rating					

*Denotes statistically significant difference

## Discussion

This work explored the effect of supporting the hemiparaetic arm proximally between the shoulder and elbow. The aim of the study was to improve reaching performance by reducing the magnitude of abnormal flexion synergies associated with exerting effort at the shoulder to lift the arm. The results point to this being the case, with most participants showing improved performance in a virtual reaching task with Movement Time to target showing the most improvement with Throughput, Path Efficiency, and the amount of Overshoots showing more modest improvement with support in some to most participants.

### Mitigated abnormal flexion synergy

By providing arm support, the overall effort to lift the arm was reduced, which has been shown to reduce the eliciting of unwanted flexor activation in individuals with hemiparesis [9–12,14]. While using arm support, the large majority of subjects showed reduced muscle activity in the anterior deltoid and trapezius, indicating a reduction in effort to lift the arm (Table 2). This is coupled with a reduction in bicep activity, particularly when reaching to targets requiring more extension. This demonstrates that arm support can reduce unwanted flexion of the elbow allowing for more controlled extension of the arm. This, combined with the observed improvements in reaching, point to a reduction in the abnormal flexion synergy, allowing most participants to better express what retained voluntary arm extension they had to reach targets more swiftly and for some, more efficiently and with less Overshoot (Fig 3, Fig 5).

Participants still exhibited significant weakness in their impaired arm, impacting the number of types of targets they could reach. Fig 2 shows that while some targets were much more reachable with support (primarily those requiring a greater amount of extension which was prevented while not supported) for the most part, the target requiring the greatest amount of extension was still out of reach even with support. The weakness as a result of stroke is still a limiting factor in reaching for some subjects, but for the targets they can reach, they were able to move to faster as a result of not being hindered by abnormal flexion synergies.

### Individual participant improvements

As previously stated, most participants (five of the seven that had reduced performance in the impaired arm compared to unaffected limb) showed significant improvement in Movement Time (Fig 5). Most participants (four of seven) also improved their Path Efficiency and some (three of six) their amount of Overshoot. These improvements in endpoint accuracy are also likely a result of having a reduced flexion synergy in that they were not “fighting against themselves” and were able to exert more precise endpoint position and velocity control. These improvements lead to most participants (four of six) to have an increased Throughput when using upper arm support compared to while unsupported.

Of the individual subject improvements across performance measures, the majority improved to a point where their reaching performance while supported showed no significant difference to that of their unaffected arm (Fig 5, filled circles). This demonstrates the possibility that with upper arm support, individuals who have suffered a stroke can potentially achieve a level of reaching performance that is comparable to their pre-stroke capability.

When grouped into the categories of those who can display voluntary hand opening and those who cannot, the participants that are more impaired (but not near complete flaccidity as with Subject 6), within each category, tend to show more areas in need of improvement and are better aided by arm support. Those who are less impaired (thereby needing less assistance) show comparably less improvement. This generally indicates that those with mid-level severe to lower level moderate stroke may be able to improve their reaching with the use of upper arm support.

### Performance across subjects

Looking at average performance across subjects (Fig 6), reaching with the impaired arm without support was less capable than reaching with the unaffected limb. Of the four performance measures that were applicable to the whole cohort, only Throughput and Movement Time exhibited significant improvement with support compared to the unsupported condition. This is a result of the average individual subject performances. While most improved Movement Time, improvements in Path Efficiency and Overshoot were more variable due to each participant’s hemiparesis and resulting weakness. That said, the combined improvements across subjects were substantial enough such that average Throughput also significantly improved with support. This illustrates that both within and across participants with a range of impairment levels, upper arm support can provide significant, quantitative, and impactful improvement in reaching with the hemiparetic arm.

### Hand control

Of the nine participants, only four displayed any ability to voluntarily open the hand. For those participants, the individual participant improvements in Time On Target (Fig 5, right column) are a key sign of how upper arm support can potentially reduce abnormal flexion synergies. All three participants who exhibited Time on Target longer than their unaffected hand showed reduced impaired hand Time On Target while using support compared to unsupported and the performance of two of them was not statistically different from the unaffected limb. Reduced Time On Target indicates that they were able to open their hand to select the target faster while supported than while unsupported. While unsupported, abnormal flexion synergies close the hand, fighting against the voluntary effort to open the hand. When these are reduced by reducing the effort to lift the arm, the participants’ retained extension ability is more able to open the hand. In the two of the three subjects who showed improvement, while supported, their voluntary finger extension ability was potentially comparable to that of their unaffected hand. For some stroke survivors, the key to improving hand function isn’t to improve the ability to open the hand, but to address the unwanted flexion that fights to keep the hand closed. Similar to the pattern seen in the other performance measures, weakness can still be present, as shown in the most impaired participant in the voluntary hand opening group (Fig 5, Subject #3) who while showing improvement with support, was still a bit slower to open the supported impaired hand compared to the unaffected hand.

### User perceptions

Across subjects, significant improvements were seen in the perceived ease of reaching, controlling the position, and overall effort of using the impaired limb with support compared to without (Table 3). Ease of hand opening was assessed across all subjects and given that five of nine subjects could not voluntarily open the hand with or without support, the lack of overall improvement in that is not surprising. These results qualitatively match well with the quantified performance measures. The amount of perceived improvement is striking, with support helping to improve the ease of the reaching task comparable to a full level of difficulty (hard to moderate or moderate to easy). That this is across participants spanning a range of disability levels is particularly impactful. Not only can upper arm support provide measurable improvements, but is also felt by subjects to make things easier than while not supported.

### Impact and future work

Overall, this work demonstrates that by providing arm support at the upper arm to reduce shoulder effort, reaching, and potentially hand performance, can be improved. This compares well to prior studies of arm support [9,12,14], but indicates that where the support is placed can be closer to the body than has been previously shown. This is a key distinction, as for any worn assistive technology, size and weight play a large role in user acceptance. By supporting the upper arm instead of the lower arm distal to the elbow, the overall device can be kept smaller and lighter with matching reductions in any actuators and batteries that may be required [29]. This could lead to the development of more comfortable and acceptable worn arm support device.

That said, arm support will not completely restore arm function after stroke. The effect of stroke is two-pronged–one is the abnormal flexion synergy that is potentially reduced with arm support, the other is the weakness due to hemiparesis. The first issue can be addressed with support and can reduce the effect of the second by allowing weak, though still functionally relevant capability to be expressed, but weakness remains an issue, even with support.

The positive aspect of this is that restoring arm and finger extension is fairly straight forward using either mechanical assistance [35,36] or electrical stimulation [37–39]. Systems for these assistance modalities are already on the market and could be coupled with upper arm support to improve their effectiveness. By reducing the abnormal flexion synergies, extension assistance systems will have less resistance to overcome and will be better able to improve reaching function. Depending on the nature of a potential user’s stroke, they may only need upper arm support if their retained voluntary extension is enough to be functionally relevant. For others with more substantial weakness, they may need a combined system that combines upper arm support with a currently available hemiparesis assistance device. The utility of such a combined system will be the topic of future efforts following up on this work.

### Limitations

While the results are possibly compelling, several limitations exist in the current study. The most significant is the number of subjects who do not have voluntary hand opening. With only four of nine subjects able to open the hand (with or without support) it makes for an admittedly small cohort with conclusions on the impact of upper arm support on hand opening limited to individual subject performance. This also affected the participant perceptions of hand ability on the URS evaluation. The overall cohort was also relatively small–large enough to show feasibility, but population conclusions on some performance measures remained too variable to demonstrate significant differences between supported and unsupported performance. The long-term learning effects or any potential therapeutic impacts of chronic use of upper arm support were also not explored. These will be addressed in future studies based on this early preliminary work.

## Conclusion

Stroke is a common cause of disability and can have a substantial impact on the ability to functionally use the impaired arm and hand. The effects of a stroke are primarily two-fold–weakness and abnormal muscle synergies that produce unwanted muscle contractions during particular movements. One common abnormal synergy is the shoulder-elbow flexion synergy where effort to raise the arm at the shoulder can result in flexion of the elbow, wrist, and fingers. Supporting the arm to reduce evoking this unwanted action has been shown in previous studies to improve arm and hand performance, but many of the means to provide said support are not wearable or easily portable. This work explored the impact of upper arm support on hand and arm performance in an effort to demonstrate that supporting the arm more proximal to the body can offer comparable benefits to forearm support.

Nine participants were assessed using a virtual reaching task while their impaired arm was supported by a robot arm, while unsupported, and while using their unaffected limb. Performance was quantified using published performance measures and participant perceptions of the ease of reaching was recorded. Most subjects showed improvements in reaching while supported compared to unsupported in Movement Time with fewer also improving their Throughput, Path Efficiency, and Overshoots. For those subjects who could voluntarily open their hand, most reduced the time it took to open their hand while supported compared to while unsupported. Many of the performance measures while supported were not significantly different to those while using the unaffected limb, indicating that reaching with upper arm support may potentially match that of the impaired arm’s pre-stroke performance. Analysis of the muscle activity of the arm showed that upper arm support does reduce the effort to lift the arm and help to reduce biceps activity in most subjects, allowing for easier reaches for targets requiring arm extension. User perceptions of using arm support were positive and indicated significant improvements in the ease of reaching with the impaired limb. While upper arm support can address the abnormal flexion synergies that can commonly occur after stroke, weakness due to hemiparesis remains. Future systems combining upper arm support with mechanical or electrical stimulation arm and finger extension devices may help to restore function in the hemiparetic arm more effectively than when used without support.
